# Editorial: Interplay of immunothrombosis and thromboinflammation in health and disease

**DOI:** 10.3389/fcvm.2026.1831697

**Published:** 2026-04-13

**Authors:** Krystin Krauel, Klytaimnistra Kiouptsi, Giulia Pontarollo, Carsten Deppermann, Christoph Reinhardt

**Affiliations:** 1 Department of Cardiology, Haemostaseology, and Medical Intensive Care, Medical Centre Mannheim, Medical Faculty Mannheim, Heidelberg University, Mannheim, Germany; 2 German Center for Cardiovascular Research (DZHK), Partner Site Heidelberg/Mannheim, Mannheim, Germany; 3 Center for Thrombosis and Hemostasis (CTH), University Medical Center of the Johannes Gutenberg-University Mainz, Mainz, Germany; 4 Clinic for Gastroenterology und Hepatology, University Hospital Zürich, Zurich, Switzerland; 5 Research Center for Immunotherapy, University Medical Center Mainz, Johannes Gutenberg-University, Mainz, Germany; 6 German Center for Cardiovascular Research (DZHK), Partner Site Rhine-Main, Mainz, Germany

**Keywords:** coagulation, immunothrombosis, inflammation, thromboinflammation, thrombosis

## Abstract

**Figure:**
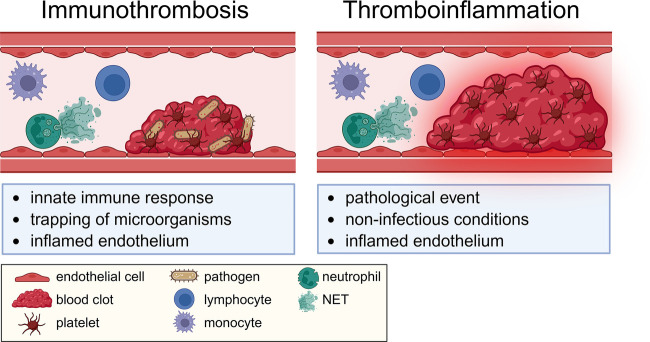

Immunothrombosis and thromboinflammation are increasingly recognized across clinical disciplines. Although the concepts have both emerged over a decade ago (1–4), significant gaps remain in our understanding of how host defense and sterile inflammation are linked to thrombus formation (5). Immunothrombosis is an innate immune response that triggers the coagulation system to cause the formation of thrombi inside blood vessels of infected individuals. This physiological thrombus formation protects the host from invading pathogens and helps prevent the dissemination of blood-borne microorganisms. In contrast, thromboinflammation is a pathologic state usually occurring under non-infectious conditions, in which inflamed endothelium promotes coagulation and immune activation leading to thrombus formation. This pathological thrombosis shares similar cellular and molecular mechanisms and occurs in large veins, arteries, and microvessels.

The present Research Topic deals with the innate immune sensing engaged in venous thromboembolism (Wu et al.), the role of factors triggering (Rapon et al.) or modulating (Bai et al.) thromboinflammatory processes, the identification of thromboinflammatory biomarkers (Chen et al., Li et al.), research methods that enable the visualization of thrombi (Kranz et al.), and treatment strategies of thrombotic complications (Qiu et al.).

Clearly, the integration of inflammatory stimuli in a cell-specific manner orchestrates immunothrombotic responses. The review by Wu et al. provides an overview of how the NLRP3 inflammasome can contribute to the development of venous thrombosis. It describes the NLRP3 inflammasome signaling pathway, summarizes how the resulting secretion of pro-inflammatory cytokines, predominantly by macrophages, activates other leukocytes, platelets, and endothelial cells, and gives an overview of the relevant pathomechanisms of venous thrombosis. Importantly, Wu et al. summarize the emerging roles of NLRP3 inhibitors in antithrombotic therapies.

A so far neglected aspect in thromboinflammation is genetic predisposition. Bai et al. review the controversial role of the DNA demethylase Ten-Eleven Transformation 2 (TET2), a recognized factor in hematological malignancies and venous thromboembolism. TET2 has been shown to affect platelet number and function, endothelial cell function, vascular smooth muscle cell differentiation, coagulation factor expression, as well as inflammatory responses and NETosis. The authors discuss the link between TET2 mutations and venous thromboembolism risk and the suitability of TET2 as a potential therapeutic target.

In addition to genetic predisposition and microbial stimuli, snake venoms can also trigger the formation of intravascular thrombi. As a consequence of snake bites, thromboinflammatory complications are frequently observed. The venoms of South and Middle American *Bothrops* snakes in tropical regions activate blood clotting. The review article by Rapon et al. provides an overview of the toxic effects of *Bothrops* venoms, including the activation of innate immune receptors and the consumption of coagulation factors and platelets, leading to thrombocytopenia and hemorrhage. The article focuses on the procoagulant and cell type-specific inflammatory effects of *Bothrops* venoms and the rare thrombotic complications that can result.

The development of diagnostic markers and risk stratification for the occurrence of arterial and venous thrombotic events in cardiovascular diseases is steadily advancing. Chen et al. analyze the utility of platelet CD147 expression as a biomarker for assessing atherosclerotic plaque characteristics in a population with coronary heart disease at moderate altitude. CD147 is a transmembrane glycoprotein present on the platelet surface and in alpha granules. Comparing a cohort of stable angina with a cohort of acute coronary syndrome patients revealed that elevated platelet CD147 expression, measured by flow cytometry, correlated with plaque instability under moderate hypoxic conditions.

The retrospective cohort study by Li et al. identified elevated neutrophil count, elevated uric acid level, and reduced platelet-to-lymphocyte ratio as inflammatory biomarkers for acute pulmonary embolism risk and disease severity in lung cancer patients. Thus, advances in precision medicine will improve future treatment of thromboinflammatory diseases.

The use of medical technology devices and related thrombolytic strategies across various disease settings is an important field in which thromboinflammation remains a limitation for therapeutic outcomes. For histological analysis of blood clots formed during extracorporeal membrane oxygenation treatment, Kranz et al. developed a new method that enables cross-sectional microtome cutting through the membrane lung facilitated by increased mechanical stability provided by polymer embedding. The case report by Qiu et al. describes how divergent thrombolytic strategies during venoarterial extracorporeal membrane oxygenation in patients with pulmonary embolism-induced cardiac arrest may result in different clinical outcomes. Such case reports are instrumental to optimizing treatment strategies.

Although immunothrombosis and thromboinflammation share common cellular and molecular mechanisms, our understanding of environmental and genetic factors that predispose the transition from physiologic immune response to vascular pathology is limited (6, 7). There are increasing numbers of sequencing studies and correlations of clinical laboratory parameters from studies of thromboinflammatory diseases. This leads to the discovery of disease-specific biomarkers (8). However, to identify functional biomarkers that are predictive in clinical settings of such disease entities, interventional studies in large animal models are required. The closure of this translational gap, coupled with new functional biomarkers and clinical studies, will foster precision medicine for the tailored treatment of thromboinflammatory diseases.
